# CNS tumors with *PLAGL1*-fusion: beyond *ZFTA* and *YAP1* in the genetic spectrum of supratentorial ependymomas

**DOI:** 10.1186/s40478-023-01695-7

**Published:** 2024-04-05

**Authors:** Arnault Tauziède-Espariat, Yvan Nicaise, Philipp Sievers, Felix Sahm, Andreas von Deimling, Delphine Guillemot, Gaëlle Pierron, Mathilde Duchesne, Myriam Edjlali, Volodia Dangouloff-Ros, Nathalie Boddaert, Alexandre Roux, Edouard Dezamis, Lauren Hasty, Benoît Lhermitte, Edouard Hirsch, Maria Paola Valenti Hirsch, François-Daniel Ardellier, Mélodie-Anne Karnoub, Marie Csanyi, Claude-Alain Maurage, Karima Mokhtari, Franck Bielle, Valérie Rigau, Thomas Roujeau, Marine Abad, Sébastien Klein, Michèle Bernier, Catherine Horodyckid, Clovis Adam, Petter Brandal, Pitt Niehusmann, Quentin Vannod-Michel, Corentin Provost, Nicolas Menjot de Champfleur, Lucia Nichelli, Alice Métais, Cassandra Mariet, Fabrice Chrétien, Thomas Blauwblomme, Kévin Beccaria, Johan Pallud, Stéphanie Puget, Emmanuelle Uro-Coste, Pascale Varlet, RENOCLIP-LOC

**Affiliations:** 1 Department of Neuropathology, GHU Paris-Psychiatrie Et Neurosciences, Sainte-Anne Hospital, 1, Rue Cabanis, 75014 Paris, France; 2 Department of Pathology, Toulouse University Hospital, Toulouse, France; 3 Cancer Research Center of Toulouse (CRCT), INSERM U1037, Toulouse, France; 4 Department of Neuropathology, Institute of Pathology, University Hospital Heidelberg, Heidelberg, Germany; 5 Clinical Cooperation Unit Neuropathology, German Cancer Research Center DKFZ, German Consortium for Translational Cancer Research (DKTK), Heidelberg, Germany; 6 Paris-Sciences-Lettres, Curie Institute Research Center, INSERMU830 Paris, France; 7 Laboratory of Somatic Genetics, Curie Institute Hospital, Paris, France; 8 Department of Pathology, Dupuytren University Hospital, Limoges, France; 9 Radiology Department, AP-HP, Raymond Poincaré Hospital, 92380 Garches, France; 10 Pediatric Radiology Department, AP-HP, Hôpital Universitaire Necker-Enfants Malades, France, and Université de Paris, INSERM ERL UA10, INSERM U1163, Institut Imagine, F-75015 Paris, France; 11 Department of Neurosurgery, GHU Paris-Psychiatrie Et Neurosciences, Sainte-Anne Hospital, Paris, France; 12 Department of Pathology, Strasbourg Hospital, Strasbourg, France; 13 Department of Neurology, Strasbourg Hospital, Strasbourg, France; 14 Radiology 2 Department, Strasbourg University Hospital, Hautepierre Hospital, Strasbourg, France; 15 Engineering Science, Computer Science and Imaging Laboratory (ICube), Integrative Multimodal Imaging in Healthcare, UMR 7357, University of Strasbourg-CNRS, Strasbourg, France; 16 Department of Pediatric Neurosurgery, Lille University Hospital, 59000 Lille, France; 17 Institute of Pathology, Centre de Biologie Pathologie, Lille University Hospital, 59000 Lille, France; 18 Sorbonne Université, AP-HP, Institut du Cerveau - Paris Brain Institute - ICM, Inserm,, CNRS, Hôpitaux Universitaires La Pitié Salpêtrière - Charles Foix, Service de Neuropathologie, 75013 Paris, France; 19 Department of Pathology, Gui de Chauliac Hospital, 34295 Montpellier, France; 20 Department of Neurosurgery, Gui de Chauliac Hospital, 34295 Montpellier, France; 21 Department of Pathology, Jean Minjoz Hospital, Besançon, France; 22 Department of Pediatric Oncology, Jean Minjoz Hospital, Besançon, France; 23 Department of Pathology, Foch Hospital, Paris, France; 24 Department of Neurosurgery, Foch Hospital, Paris, France; 25 Department of Pathology, Bicêtre Hospital, 94275 Le Kremlin-Bicêtre, France; 26 Section for Cancer Cytogenetics, Institute for Cancer Genetics and Informatics, Oslo University Hospital, Oslo, Norway; 27 Department of Oncology, Oslo University Hospital-The Norwegian Radium Hospital, Oslo, Norway; 28 Devision of Cancer Medicine, Oslo University Hospital, Oslo, Norway; 29 Department of Pathology, Oslo University Hospital, Oslo, Norway; 30 Radiology Department, Lille University Hospital, 59000 Lille, France; 31 Department of Radiology, GHU-Paris-Psychiatrie Et Neurosciences, Hôpital Sainte Anne, 75014 Paris, France; 32 Department of Radiology, Gui de Chauliac Hospital, 34295 Montpellier, France; 33 Department of Neuroradiology, Sorbonne Université, AP-HP, Institut du Cerveau - Paris Brain Institute - ICM, Inserm, CNRS, Hôpitaux Universitaires La Pitié Salpêtrière - Charles Foix, 75013 Paris, France; 34 Institute of Psychiatry and Neuroscience of Paris (IPNP), Université Paris Cité, INSERM U1266, Imabrain Team, 75014 Paris, France; 35 Department of Pediatric Neurosurgery, Necker Hospital, APHP, Université Paris Descartes, Sorbonne Paris Cité, Paris, France; 36 Department of Neurosurgery, La Martinique Hospital, Fort-de-France, France; 37 Université Paul Sabatier, Toulouse III, Toulouse, France

**Keywords:** Ependymoma, PLAGL1, Subependymoma, DNA-methylation

## Abstract

A novel methylation class, “neuroepithelial tumor, with *PLAGL1* fusion” (NET-PLAGL1), has recently been described, based on epigenetic features, as a supratentorial pediatric brain tumor with recurrent histopathological features suggesting an ependymal differentiation*.* Because of the recent identification of this neoplastic entity, few histopathological, radiological and clinical data are available. Herein, we present a detailed series of nine cases of *PLAGL1*-fused supratentorial tumors, reclassified from a series of supratentorial ependymomas, non-*ZFTA/*non*-YAP1* fusion-positive and subependymomas of the young. This study included extensive clinical, radiological, histopathological, ultrastructural, immunohistochemical, genetic and epigenetic (DNA methylation profiling) data for characterization. An important aim of this work was to evaluate the sensitivity and specificity of a novel fluorescent in situ hybridization (FISH) targeting the *PLAGL1* gene. Using histopathology, immunohistochemistry and electron microscopy, we confirmed the ependymal differentiation of this new neoplastic entity. Indeed, the cases histopathologically presented as “mixed subependymomas-ependymomas” with well-circumscribed tumors exhibiting a diffuse immunoreactivity for GFAP, without expression of Olig2 or SOX10. Ultrastructurally, they also harbored features reminiscent of ependymal differentiation, such as cilia. Different gene partners were fused with *PLAGL1*: *FOXO1, EWSR1* and for the first time *MAML2*. The *PLAGL1* FISH presented a 100% sensitivity and specificity according to RNA sequencing and DNA methylation profiling results. This cohort of supratentorial *PLAGL1*-fused tumors highlights: 1/ the ependymal cell origin of this new neoplastic entity; 2/ benefit of looking for a PLAGL1 fusion in supratentorial cases of non-*ZFTA/*non*-YAP1* ependymomas; and 3/ the usefulness of *PLAGL1* FISH.

**Supplementary Information:**

The online version contains supplementary material available at 10.1186/s40478-023-01695-7.

## Introduction

Ependymomas (EPN) are glial neoplasms that affect mainly children and young adults. New insights in the genomic and epigenetic landscape of EPN have led to the identification of different groups, according to their anatomic location (supratentorial, posterior fossa and spinal) [25]. Three subgroups have been identified among supratentorial tumors (ST-EPN): subependymomas; EPN, *YAP1* fusion-positive; and EPN, *ZFTA* fusion-positive (as specified by the World Health Organization’s (WHO-2021) classification) [3, 24–26]. In a recent study, the methylation classifier based on Forest plot random classification identified a novel methylation class (MC) characterized by the presence of a *PLAGL1* (*pleomorphic adenoma gene-like 1*) gene fusion [29]. *PLAGL1* is one of the PLAG family genes (which also include *PLAG1* and *PLAGL2*) initially implicated in the tumorigenesis of pleomorphic adenomas, and several other cancers [14, 17, 19, 38]. In the central nervous system (CNS), most neoplasms presenting a *PLAGL1* fusion have initially been diagnosed as ependymomas in the supratentorial area of children and young adults (median age of 6.2 years old, ranging from 0 to 30) [29]. The majority of cases presented histopathological and immunohistochemical findings (GFAP immunopositivity without expression of Olig2 or SOX10) suggestive of an ependymal differentiation or subependymoma-like features (microcystic changes) [29]. However, *PLAGL1* fusions seem to produce other morphological patterns, including glial, glioneuronal, embryonal and even epithelial characteristics [29]. Because of this morphological spectrum, the terminology “neuroepithelial tumor, with *PLAGL1*-fusion” (NET-PLAGL1) was preferred while awaiting additional studies. Moreover, because of this recent description, very few data concerning clinical, and radiological data as well as outcome, are available in the literature [29]. In this study, we performed a clinico-pathological and molecular analysis (which included DNA-methylation profiling) for 9 new cases of NET with *PLAGL1* fusion, to more suitably characterize these tumors.

## Materials and methods

### Study design, patients, data collection

This study included patients diagnosed between January 1, 1996 and September 30, 2022 with 1) a supratentorial (ST) ependymoma without a *ZFTA* or *YAP1* fusion, or 2) subependymoma of young patients less than 40 years old (the peak incidence of classical subependymomas being defined as occurring in patients aged 40 to 84 years in the current WHO classification [21]), determined by fluorescence in situ hybridization (FISH) and RNA-sequencing analyses (techniques previously described in [24]), from GHU-Paris, Sainte-Anne Hospital’s archives and the French National Neuropathological Network (RENOCLIP-LOC).

Epidemiological data (gender and age at diagnosis) concerning the tumor and treatment-related data (location of tumor and extension, extent of resection, relapses and complementary treatments) were retrospectively analyzed. The extent of the initial resection was assessed by magnetic resonance imaging (MRI) or computed tomography performed after surgery. Written informed consent to participate in this study was provided by the participants or participants' legal guardian. This study was reviewed and approved by our local Ethic Committee.

### Statistical analyses

Unadjusted survival curves for overall survival (OS) and progression-free survival (PFS) were plotted by the Kaplan–Meier method, using log-rank tests to assess significance for group comparison. A p-value of less than 0.05 was considered significant. Statistical analyses were performed using JMP software (version 17.0.0, SAS Institute Inc, Cary, USA). We pooled our data with that of previously reported cases of NET-PLAGL1 [29], ST non-*RELA, ZFTA*-fused ependymomas [32, 37, 44] and compared them with data from known ependymomas, *ZFTA::RELA* fusion-positive, and other histopathological differential diagnoses such as ependymomas, *YAP1* fusion-positive, CNS tumors with *BCOR* internal tandem duplication*,* and astroblastomas, *MN1-*altered [2–4, 6, 8, 11, 12, 16, 18, 24, 27, 30, 34, 39, 42].

### Central radiological review

The French Neuroimaging-RENOCLIP consortium, composed of neuroradiologist experts in the field of neurooncology, conducted a comprehensive re-evaluation of the imaging cases. This review, organized by a neuroradiologist (ME), involved the participation of 8 neuroradiologists (NB, VDR, QVM, NM, CP, LN, FDA, ME). The examination was centralized and focused on the imaging cases within the discussed cohort. The following features were evaluated on preoperative MRIs: location, size, signal in T1 and T2 weighted sequences, as well as on susceptibility imaging, on diffusion weighted imaging, and on perfusion weighted imaging, when available, presence of enhancement, cysts, and necrosis.

### Central histopathological review

The central pathology review was performed conjointly by two neuropathologists (ATE and PV). Samples were stained with haematoxylin-phloxin-saffron (HPS) according to standard protocol. For each case, the following pathological features were researched: diffuse or circumscribed growth pattern (based on histopathologically-entrapped neurons in the tumor and by using neurofilament staining), microvascular proliferation, tumoral necrosis, calcification, dysmorphic ganglion cells, perivascular mononuclear inflammatory infiltrates, eosinophilic granular bodies, Rosenthal fibers, pseudorosettes, embryonal components, clear cell components, microcystic changes, fibrillary matrix architecture, and siderophages. The mitotic index was monitored using 10 high-power fields (HPF), which corresponded to 2.3 mm^2^ on our microscope and was jointly counted by two neuropathologists in a hot-spot area. Integrated diagnoses were performed in accordance with the current WHO classification.

### Immunohistochemistry

Unstained 3-μm-thick slides of formalin-fixed, paraffin-embedded (FFPE) tissues were obtained and submitted for immunostaining using an automated stainer (Dako Omnis, Glostrup, Denmark). The following primary antibodies were used: Glial Fibrillary Acidic Protein (GFAP) (1:200, clone 6F2, Dako, Glostrup, Denmark), Olig2 (1:500, clone OLIG2, Sigma-Aldrich, Saint-Louis, USA), SOX10 (1:50, clone A-2, Diagomics, Blagnac, France), neurofilament (1:100, clone NF70, Dako, Glostrup, Denmark), NeuN (1:1000, clone A60, Sigma-Aldrich, Saint-Louis, USA), synaptophysin (1:150, clone Synap, Dako, Glostrup, Denmark), chromogranin A (1:200, clone LK2 H10, Diagnostic Biosystem, Pleasanton, USA), EMA (1:200, clone GM008, Dako, Glostrup, Denmark), L1CAM (1:500, clone UJ127.11, Sigma-Aldrich, Saint-Louis, USA), NFκB (1:6000, clone D14E12, Cell Signaling Technology, Danvers, USA), H3K27me3 (1:2500, polyclonal, Diagenode, Liege, Belgium), and Ki-67 (1:200, clone MIB-1, Dako, Glostrup, Denmark). External positive and negative controls were used for all antibodies and staining. MIB-1 labeling index was jointly estimated by two neuropathologists in a hot-spot area.

### Detection of *PLAGL1* fusion/rearrangement by RNA-Sequencing and FISH analysis

RNA was isolated from FFPE tissue with sufficient tumoral density. RNA was extracted using the High Pure FFPET RNA Isolation Kit (catalogue # 06650775001 Roche diagnostics GmbH) according to the manufacturer’s instructions. The RNA concentrations were measured on a Qubit 4 Fluorometer (# Q33238, Thermo Fisher Scientific) with the Invitrogen Qubit RNA BR Kit (# Q10210, Thermo Fisher Scientific). The percentage of RNA fragments > 200 nt (fragment distribution value; DV200) was evaluated by capillary electrophoresis (Agilent 2100 Bioanalyzer). DV200 > 30% was required to process the next steps in the analysis. NGS-based RNA sequencing was performed using the Illumina TruSight RNA Fusion Panel on a Nextseq550 instrument according to the manufacturer’s instructions (Illumina, San Diego, CA, USA). This targeted RNA sequencing panel covers 507 fusion-associated genes, to assess the most recognized cancer-related fusions. The TruSight RNA fusion panel gene list is available at https://www.illumina.com/content/dam/illumina-marketing/documents/products/gene_lists/gene_list_trusight_rna_fusion_panel.xlsx. 7,690 exonic regions are targeted with 21,283 probes. Libraries were prepared according to the Illumina instructions for the TruSight RNA fusion Panel kit. STAR_v2.78a and Bowtie software were used to produce aligned readings in relation to the Homo Sapiens Reference Genome (UCSC hg19). Manta v1.4.0, Tophat2 and Arriba v2.1.0 tools were used for fusion calling.

FISH assessment was performed on interphase nuclei in paraffin-embedded tissue (4 µm), as previously described [14]. *PLAGL1* FISH was performed using a break-apart custom SureFISH probe and hybridized according to the manufacturer's recommendations for SureFISH probes (Agilent Technologies, Santa Clara, CA) covering 3’PLAGL1 and 5’PLAGL1 regions on 11q13.1 (G110996R-8, labelled with 5-TAMRA and G110996g-8 labelled with 5-fluorescein-deoxyuridine triphosphate). Signals were scored for at least 100 non-overlapping interphase nuclei. A case was considered positive when the scored nuclei displayed a break-apart signal in at least 20% of the counted nuclei.

### Next-generation sequencing

Next-generation sequencing (NGS) was also performed in rare cases according to the Illumina NextSeq 500 protocol (Illumina, San Digeo, CA, USA).

### DNA-methylation profiling

Tumor DNA was extracted from freshly frozen tissue samples using the Qiagen DNeasy Blood & Tissue Kit (Cat NO./ID 69504) according to the manufacturer’s instructions. 500 ng of DNA were extracted from each tissue sample. DNA was sent to the Genotyping facility at the German Cancer Research Center (Heidelberg, Germany). All patient samples were analyzed using either Illumina Infinium Methylation EPIC or HumanMethylation450 BeadChip arrays in accordance with the manufacturer’s instructions. Affiliation predictions were obtained from a DNA methylation-based classification web platform for central nervous system **tumors (**https://www.molecularneuropathology.org, **version 12.8).** Next, a t-Distributed Stochastic Neighbor Embedding (t-SNE) analysis was performed and compared with the genome-wide DNA methylation profiles from the brain tumor reference cohort [10] and the previous series of NET, *PLAGL1-*fused [29]. Data were generated at the DKFZ Genomics and Proteomics Core Facility (Heidelberg, Germany) as previously described [10].

### Ultrastructural analyses

A representative section was first selected for each case from FFPE tissue stained with Hemalun Phloxin Saffron. Then, the tissue was deparaffinized and fixed one hour in glutaraldehyde. Following the dehydration process, tissues were embedded in Epon. Semi-thin Sects. (1-μm-thick slides) were stained with toluidine blue. Ultrathin Sects. (90 nm) were stained with lead citrate and uranyl acetate, then observed under an electronic microscope (JEOL JEM 1400 Flash). Analyses were performed in the Pathology Department at the Limoges University Hospital, by one neuropathologist (MD).

## Results

### Sixty percent of the 15 supratentorial subependymomas and ependymomas, non-ZFTA/non-YAP1 fused exhibited genetic and epigenetic similarities with NET-PLAGL1

**Fig. 1:**
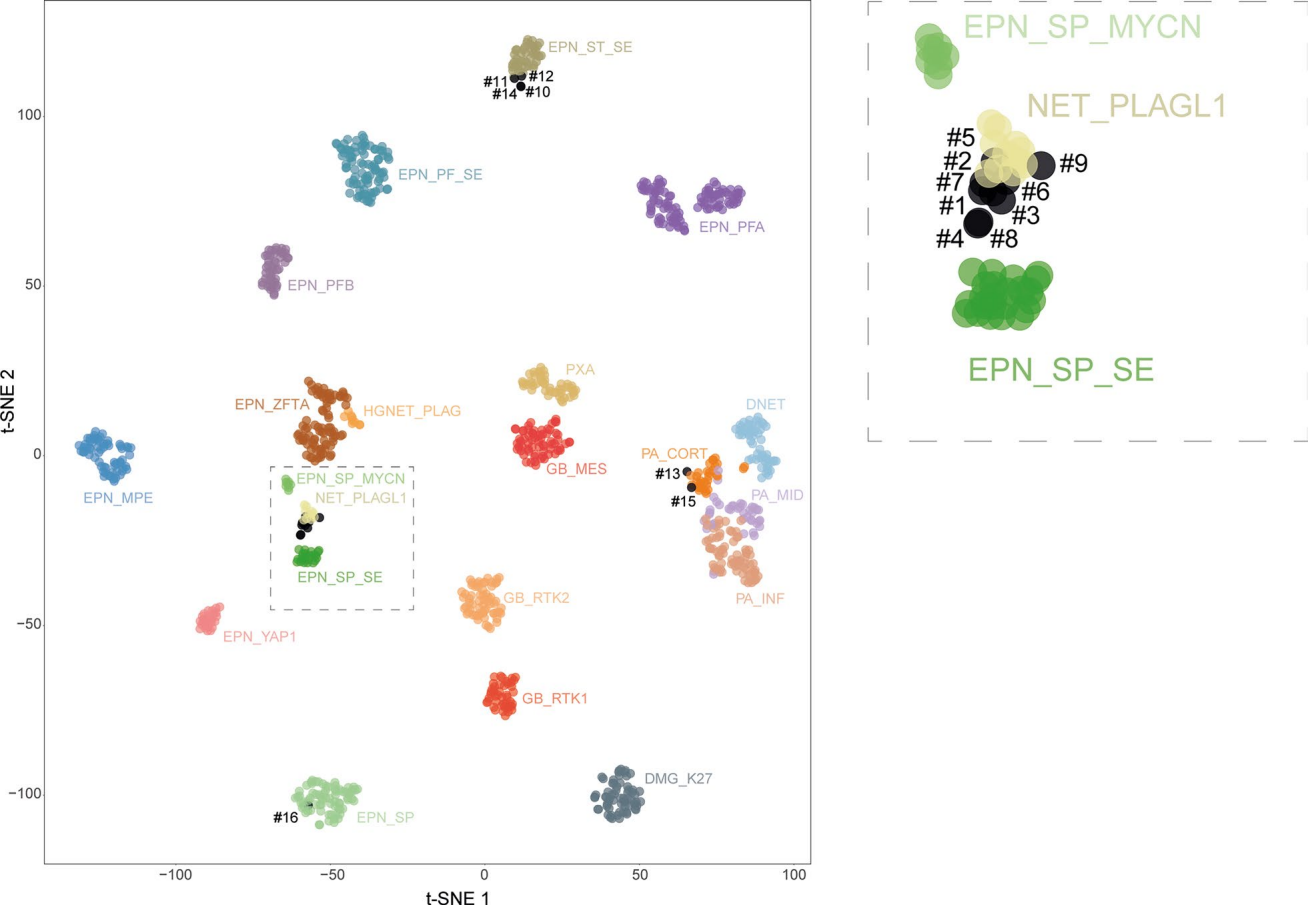
DNA methylation-based t-distributed stochastic neighbor embedding distribution. Reference DNA methylation classes (v12.5 of the DKFZ classifier): DMG_K27: diffuse midline glioma H3 K27M mutant/EZHIP overexpressing; DNET: dysembryoplastic neuroepithelial tumor; EPN_MPE: myxopapillary ependymoma; EPN_PFA: ependymoma, posterior fossa group A; EPN_PFB: ependymoma, posterior fossa group B; EPN_PF_SE: subependymoma, posterior fossa; EPN_SP_SE: subependymoma, spinal; EPN_SP: spinal ependymoma; EPN_SP_MYCN: spinal ependymoma, MYCN-amplified; EPN_ST_SE: subependymoma, supratentorial; EPN_ZFTA: ependymoma, ZFTA fusion; EPN_YAP1: ependymoma, YAP1 fusion; GB_RTK1: glioblastoma, IDH wildtype, subclass RTK1; GB_RTK2: glioblastoma, IDH wildtype, subclass RTK2; GB_MES: glioblastoma, IDH wildtype, subclass mesenchymal; HGNET_PLAG: embryonal tumor with PLAG-family amplification; NET_PLAGL1: neuroepithelial tumor with PLAGL1-fusion; PA_CORT: pilocytic astrocytoma, hemispheric; PA_INF: pilocytic astrocytoma, infratentorial; PA_MID: pilocytic astrocytoma, midline; PXA: pleomorphic xanthoastrocytoma

Alterations of the *PLAGL1* gene were found in 8/15 cases, including: *PLAGL1::FOXO1* (n = 3), *EWSR1::PLAGL1* (n = 2), *PLAGL1::EP300* (n = 1), and *PLAGL1::MAML2* (n = 1). The last case presented a chromothripsis-like pattern affecting chromosome 6. Using DNA-methylation profiling, 2/9 cases presented a high calibrated score (≥ 0.9) for the NET-PLAGL1 MC. We performed a t-SNE analysis of the whole cohort to better classify tumors with low calibrated scores (< 0.9) (Fig. 1). The six other cases harboring a *PLAGL1* alteration (with a calibrated score < 0.9) definitively clustered into the NET-PLAGL1 MC by t-SNE analysis. Interestingly, one additional case (#9) without any proven *PLAGL1* fusion clustered within the NET-PLAGL1 MC. No *PLAGL1* alteration was found in the remaining cases of the cohort (6/15 cases). In total, based on these genetic and epigenetic analyses, 9/15 tumors (60%) were diagnosed as NET-*PLAGL1* (Fig. 1). NGS failed to reveal additional mutation for tested genes in these nine cases (cf. Supplementary table 1).

### Further evidence of an ependymal differentiation in NET-PLAGL1 and identification of recurrent histopathological features

**Fig. 2:**
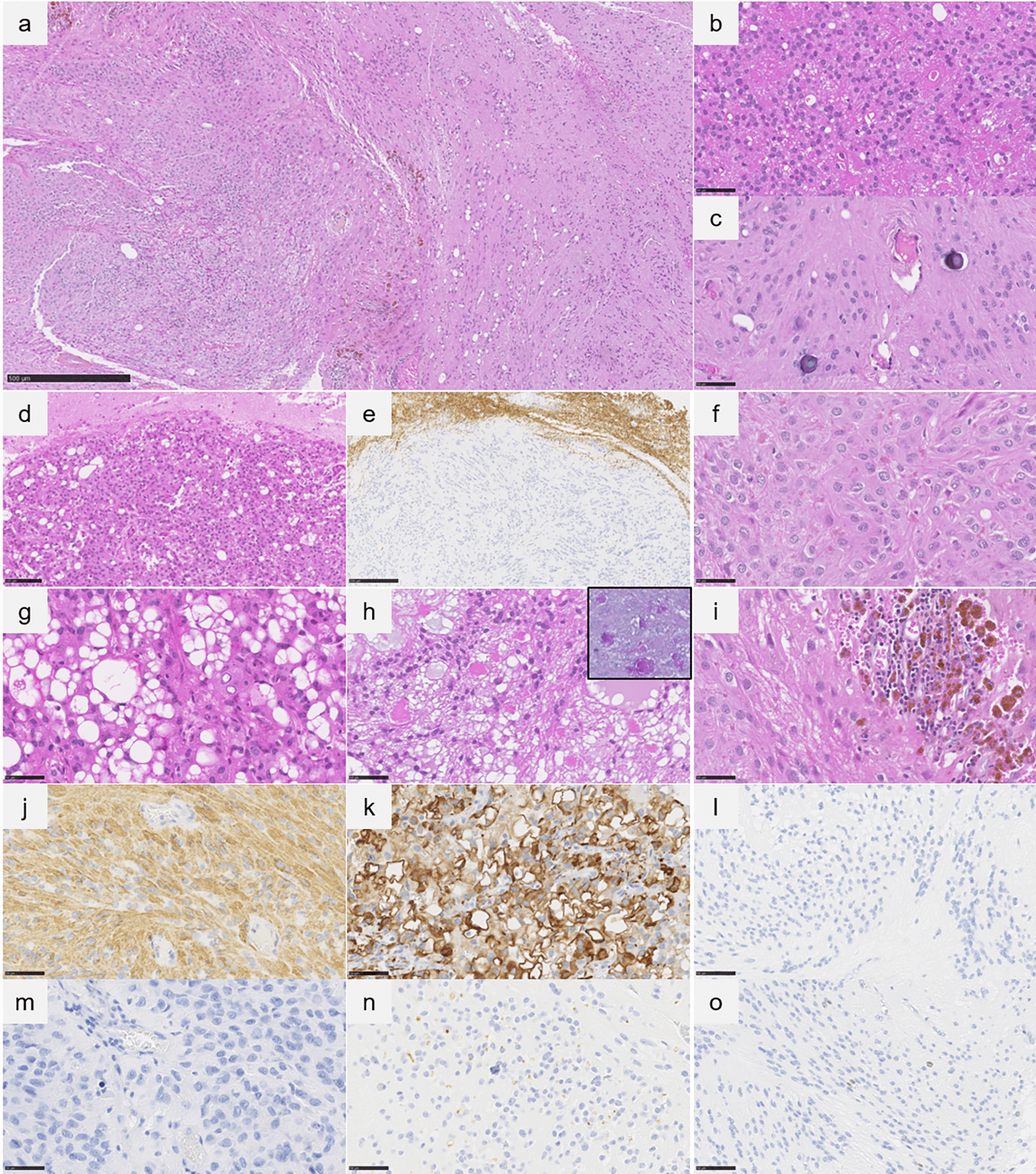
Histopathological and ultrastructural features. **a-c** An ependymal component admixed with subependymal features and microcalcifications (case #6, HPS, magnification × 60 for **a**, magnification × 400 for **b-c**). **d** Well-demarcation of the tumor from adjacent brain parenchyma (case #4, HPS, magnification × 200), confirmed using neurofilament staining (**e**, magnification × 100).** f** Monomorphous cells with small to medium-sized round nuclei and eosinophilic granular bodies (case #4, HPS, magnification × 400). **g** Frequent microcystic changes (case #6, HPS, magnification × 400). **h** Microcystic changes with myxoid substance, and eosinophilic granular bodies (case #8, HPS, magnification × 400) positive with PAS staining (case #8, insert, magnification × 400). **i** Hemorrhagic modifications with siderophages (case #4, HPS, magnification × 400). **j** Diffuse GFAP immunoexpression (case #8, magnification × 400). **k** Diffuse GFAP immunoexpression including in microcystic component (case #4, magnification × 400). **l** No immunopositivity for Olig2 (case #4, HPS, magnification × 400). **m** No immunopositivity for SOX10 (case #4, HPS, magnification × 400). **n** EMA immunoexpression with dot-like or micro-lumens (case #4, HPS, magnification × 400). **o** MIB-1 labeling index was low, ranged from 1% (case #8, HPS, magnification × 400). Black scale bars represent 500 μm (**a**), 50 µm (**b**-**c**, and **f**-**o**), 100 µm (**d**) and 250 µm (**e**) HPS: Haematoxylin Phloxin Saffron.

All NET-PLAGL1 (9/9 cases) morphologically presented an ependymal component admixed with subependymal features (Fig. 2a-c, Table 1 for main results, and Supplementary table 1 for details). All cases were well-demarcated from adjacent brain parenchyma (Fig. 2d-e), except for one (case #1) which was mostly circumscribed with a diffuse pattern at the neoplasm’s periphery. The cases were mainly composed of relatively monomorphous cells with small to medium-sized round nuclei (Fig. 2f). A few tumor cells were dystrophic. Ependymal rosettes and pseudorosettes were observed in all cases, at least focally. Clear cell (4/9 cases), papillary (1/9) and tanycytic (1/9) features were present, whereas no embryonal component was observed. Mitotic activity was low (0–2/2.3 mm^2^). In most cases (7/9), the fibrillary matrix presented frequent microcystic changes (8/9 cases), sometimes myxoid, and contained PAS-positive coarse eosinophilic granular bodies (Fig. 2g-h). A microvascular proliferation and necrosis (probably ischemic with micro-thrombi in the adjacent vessels) were present in cases three and four, respectively. Five cases presented hemorrhagic modifications with siderophages (deposition of iron pigment in macrophages suggesting a past hemorrhage) (Fig. 2i). Calcifications were present in 5/9 cases (Fig. 2c). One case presented adipocytic metaplasia. Using immunohistochemistry, all tumors except one (case #14 which presented a GFAP staining in a part of the tumor) expressed GFAP diffusely (Fig. 2j-k), and presented no or only focal immunopositivity for Olig2 and SOX10 (Fig. 2l-m). Six cases displayed EMA immunoexpression with dot-like pattern or micro-lumens (Fig. 2n). There was no expression of L1CAM and no nuclear accumulation of NFκB. Neuronal markers (synaptophysin, NeuN and chromogranin A) were negative. The MIB-1 labeling index was low, ranging from 1 to 5% (Fig. 2o).

**Table 1:** Case list of our series

Case number	Age (years-old), sex	Location	Genetic results	Epigenetic results (v12.8)	Integrated diagnoses
1	6, M	Right frontal	*PLAGL1::FOXO1*	SPINAL SUBEPENDYMOMA (0.67)	NET-PLAGL1
2	8, F	Left frontal	*PLAGL1::MAML2*	SPINAL SUBEPENDYMOMA (0.72)	NET-PLAGL1
3	37, M	Left parietal	*PLAGL1* rearrangement (chromothripsis chr. 6)	SPINAL SUBEPENDYMOMA (0.98)	NET-PLAGL1
4	38, F	Right frontal	*PLAGL1::EP300*	SUPRATENTORIAL EPN_ZFTA-FUSION POSITIVE (0.34)	NET-PLAGL1
5	9, F	Left frontal and parietal	*EWSR1::PLAGL1*	NET, PLAGL1-FUSED (1.00)	NET-PLAGL1
6	41, M	Left occipital	*EWSR1::PLAGL1*	NET, PLAGL1-FUSED (0.95)	NET-PLAGL1
7	19, M	Right temporal	*PLAGL1::FOXO1*	SPINAL SUBEPENDYMOMA (0.88)	NET-PLAGL1
8	19, F	Right temporal, parietal and occipital	*PLAGL1::FOXO1*	SUPRATENTORIAL EPN_ZFTA-FUSION POSITIVE (0.19)	NET-PLAGL1
9	13, F	Right occipital	WT	SUPRATENTORIAL SUBEPENDYMOMA (0.06)	NET-PLAGL1
10	13, F	Left occipital	WT	SPINAL, SUBEPENDYMOMA, SUBTYPE A (0.14)	ST-SE
11	36, F	Intra-ventricular	WT	SUPRATENTORIAL SUBEPENDYMOMA (0.99)	ST-SE
12	42, M	Intra-ventricular	WT	SUPRATENTORIAL SUBEPENDYMOMA (0.99)	ST-SE
13	18, M	Intra-ventricular	*C1orf194::UQCR10* and *IDH1* R172G	SUPRATENTORIAL EPN_ZFTA-FUSION POSITIVE (0.17)	LGG
14	36, M	Intra-ventricular	*C1orf194::UQCR10*	SUPRATENTORIAL SUBEPENDYMOMA (0.59)	ST-SE
15	17, M	Left frontal	*PDGFB::LRP1*	GLIOBLASTOMA MESENCHYMAL (0.07)	LGG

Chr.: chromosome; CNV: copy number variation; F: female; LGG: low-grade glioma; M: male; NA: not available; NET: neuroepithelial tumor; SE: subependymoma; ST: supratentorial; WT: wildtype

**Fig. 3:**
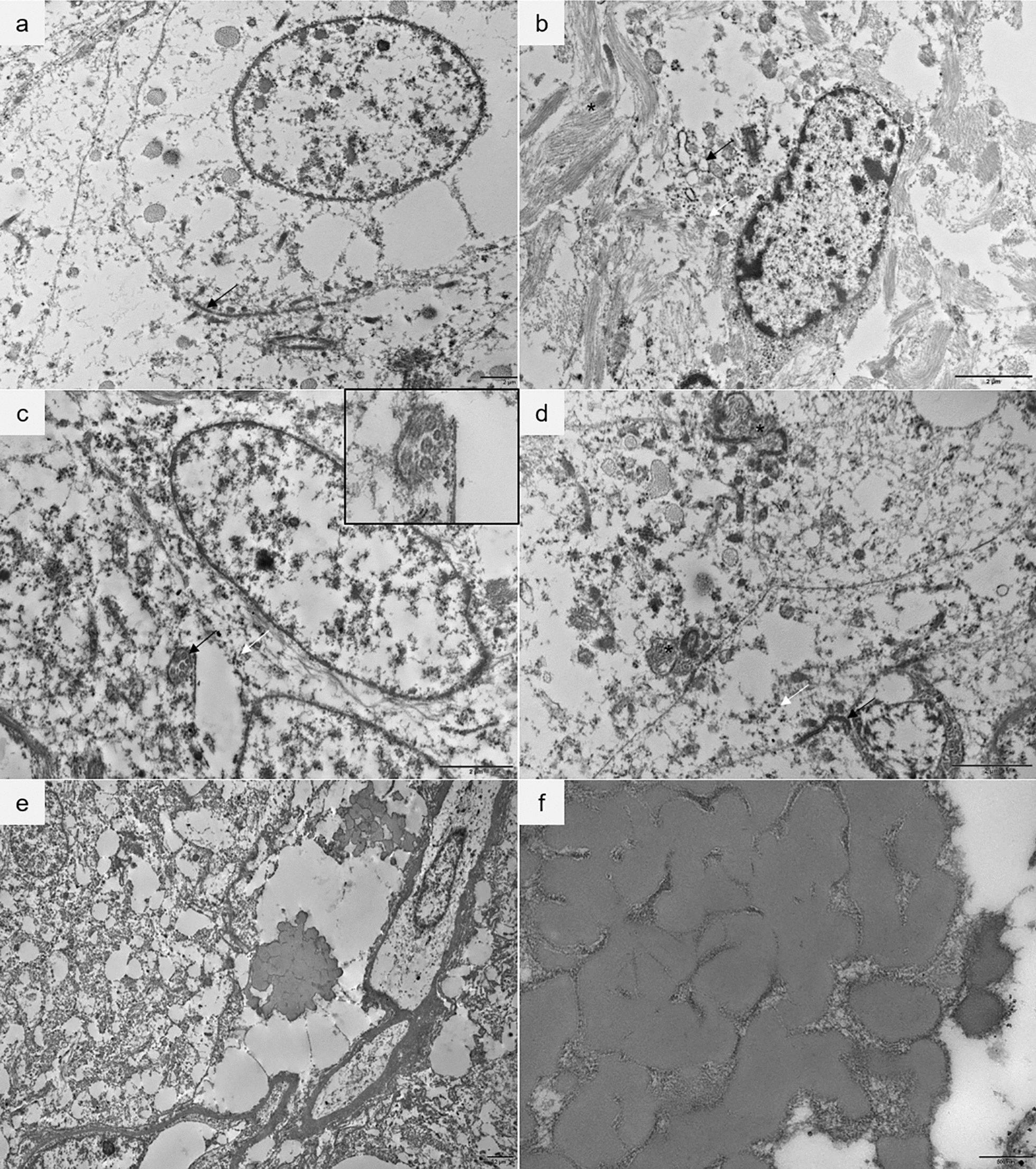
Ultrastructural findings, ultrathin sections, electron microscopy. **a** Tumor cells harboring zonula adherens (arrow).** b** Glial intermediate filaments (*) are present in the cytoplasm of the tumoral cell and in intercellular spaces. We can observe dilated cisternae of the Golgi apparatus and the smooth endoplasmic reticulum (black arrow) and numerous microtubules (white arrow). **c** Tumoral cell with an intracytoplasmic cilium in transversal section (black arrow). The white arrow shows microtubules. Insert: high magnification of the cilium. **d** Microvilli-like structures (*) are present in lumen-like spaces between the adjacent cells. The white arrow shows microtubules. **e** Extracellular eosinophilic bodies. **f** They are moderately dense, and have a shape of convolutions whose denser lines delimit guts

Ultrastructural analyses were available for seven cases (#2–6 and 8–9). All tumors presented abnormalities that clearly suggested an ependymal origin. Junctional apparatuses between the neoplastic cells, such as *zonula adherens* (Fig. 3a) or *puncta adherentia* were observed in four cases (#2–3 and 8–9). Glial intermediate filaments were present in six of seven cases (all except case #4) (Fig. 3b). All tumors presented numerous microtubules, sometimes fragmented, in the cytoplasm (Fig. 3b, c and d). Two cases (#6 and 9) harbored evident cilia in the intracellular space (Fig. 3c). In one tumor (case #3), we observed microvilli-like structures between tumor cells (Fig. 3d). Dilated cisternae of the smooth endoplasmic reticulum and the Golgi apparatus (Fig. 3b), and rectangular crystalloid bodies were seen in one case (#9). Finally, despite the lack of specificity, multiple multivesicular bodies were observed in five cases (#2–3, 5, and 8–9). There were eosinophilic bodies in cases #2, 4–6, and 8 (Fig. 3e-f). The bodies were moderately dense, and had a shape of convolutions whose denser lines delimit guts. At high magnification, we get a pitted appearance. They were extracellular and the size was variable.

### Diagnostic accuracy of newly designed PLAGL1 FISH

**Fig. 4:**
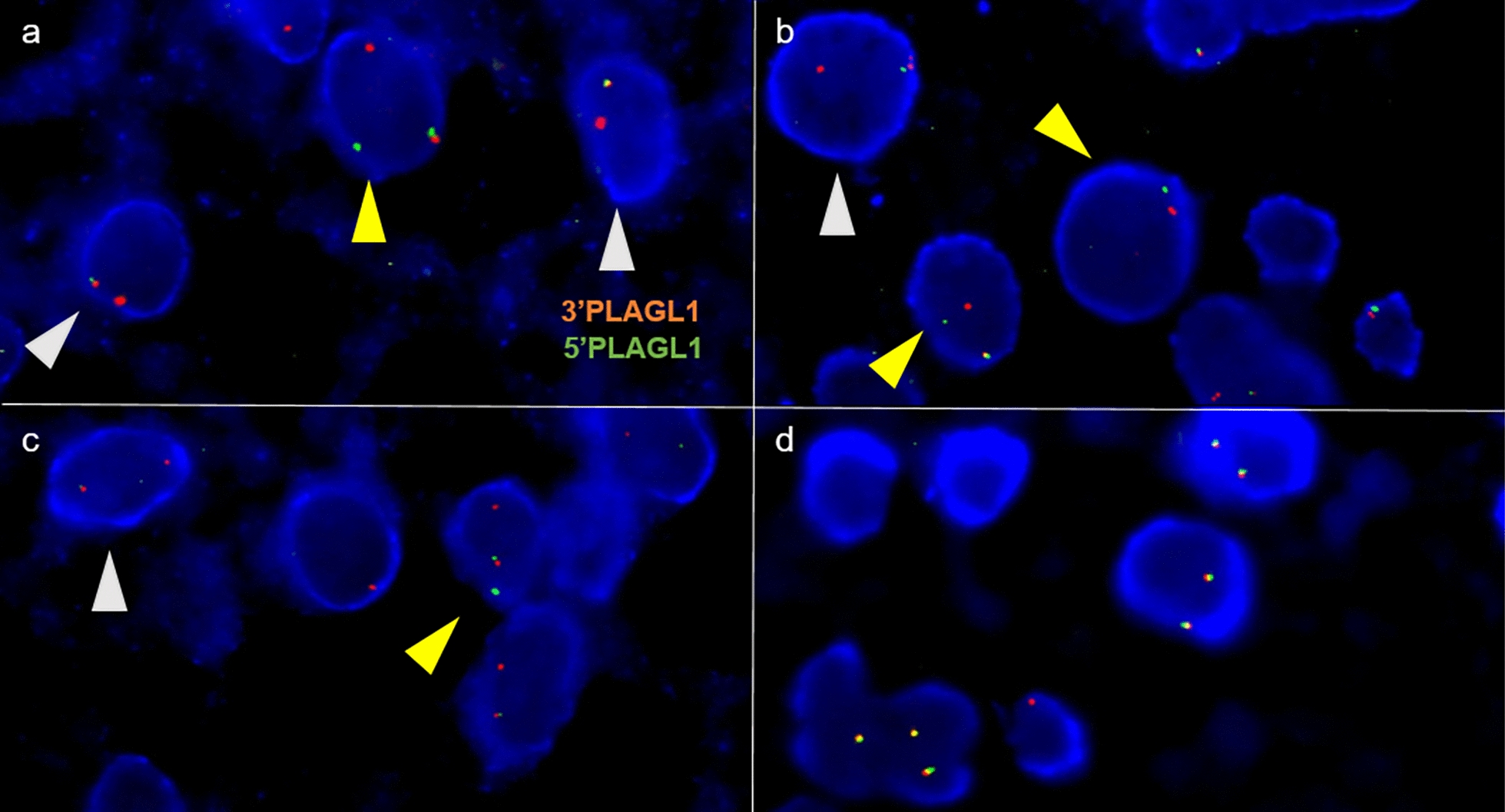
Detection of PLAGL1 rearrangements by FISH. **a-c** FISH images showing positive cases (#2–5-7) and **d** a negative case (#10) (magnifications × 1000). Representative image of a slide hybridized with a PLAGL1 Break-Apart FISH probe. In positive cases, the images show nuclei harboring a split (red and green signals, yellow arrowheads) and a fused signal or an isolated 3’PLAGL1 signal and a fused signal (grey arrowheads). For the negative case, the images show nuclei harboring two intact fused signals FISH, fluorescence in situ hybridization

FISH analyses revealed a clear rearrangement of *PLAGL1* for 6 of the 15 (40%) cases*,* which correlated with the presence of a fusion implicating the *PLAGL1* gene observed by RNA-sequencing analyses. Positive (Fig. 4a-c) and negative (Fig. 4d) cases are illustrated. Two cases (#4 and #6) with a *PLAGL1* fusion were not contributive (technical failure). The sole case presenting a *PLAGL1* rearrangement by FISH without a proven *PLAGL1* fusion by RNA-sequencing analysis, exhibited a chromothripsis of chromosome 6 and clustered with NET-PLAGL1 by t-SNE. All cases found by RNA-sequencing analysis to not have a *PLAGL1* fusion, were also shown by FISH analysis to not have a *PLAGL1* rearrangement. Consequently, the sensitivity and specificity of the *PLAGL1* FISH for the detection of the *PLAGL1*-fused NET were perfect (100%).

### Clinical and radiological characteristics of NET-PLAGL1

**Fig. 5:**
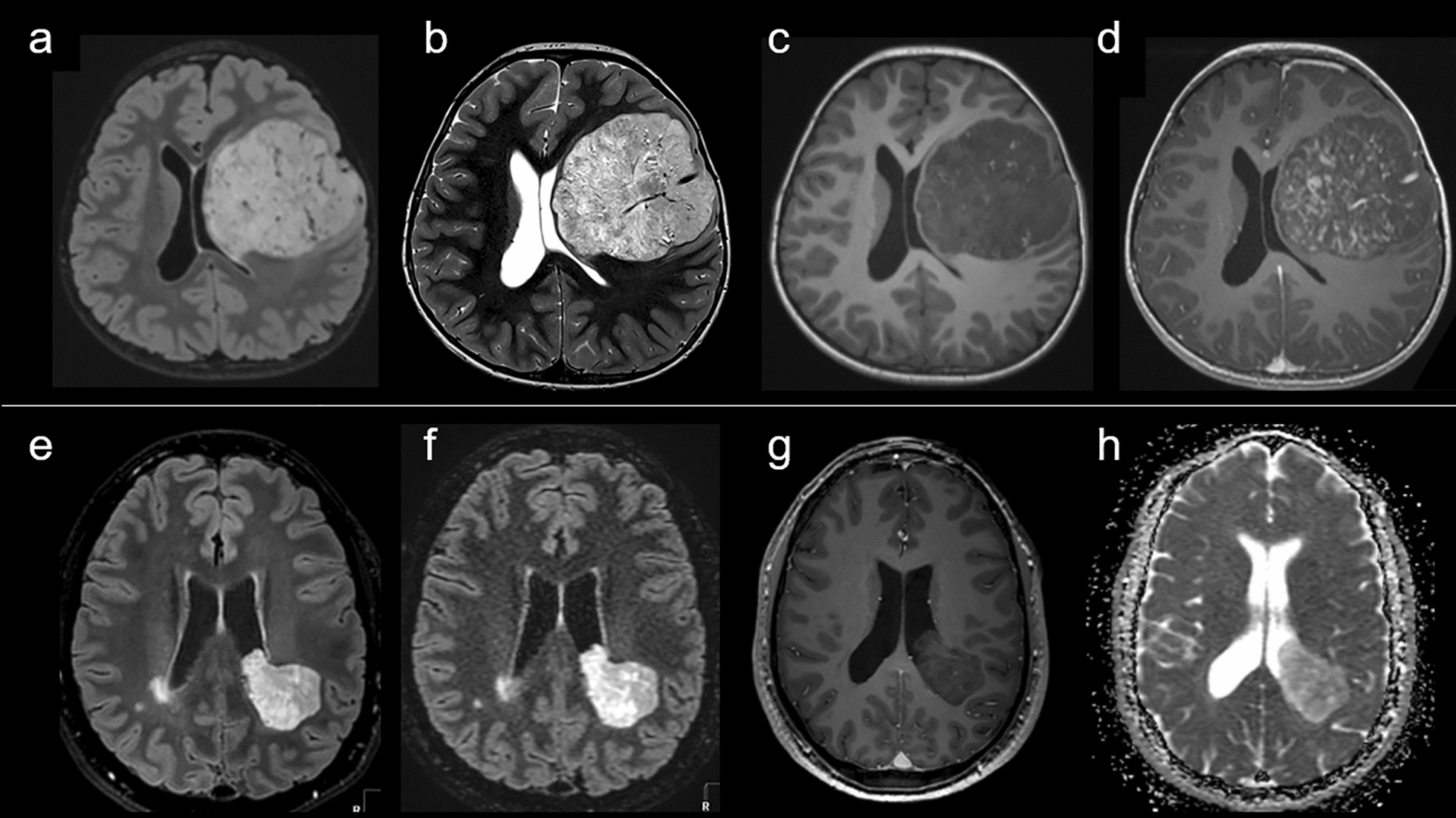
Radiological features on MRI. Illustrative image of case #2 with FLAIR-w (**a**), T2-w (**b**), T1-w (**c**) and post-contrast T1-w (**d**). MR images showing a large left frontal intra-axial brain lesion with ependymal contact. The lesion shows minimal perilesional edema, T2 hyperintensity, and a hypointense center on T1-w imaging, with subtle enhancements on post contrast imaging. Illustrative image of case #5 with FLAIR-w (**e**, **f**), post-contrast T1-w (**g**) and T2-w (**h**) axial views of MR images showing a left fronto-parietal intra-axial brain lesion with ependymal contact and extension within the ventricules. The lesion shows minimal perilesional edema, T2 hyperintensity, and barely no enhancement

**Fig. 6:**
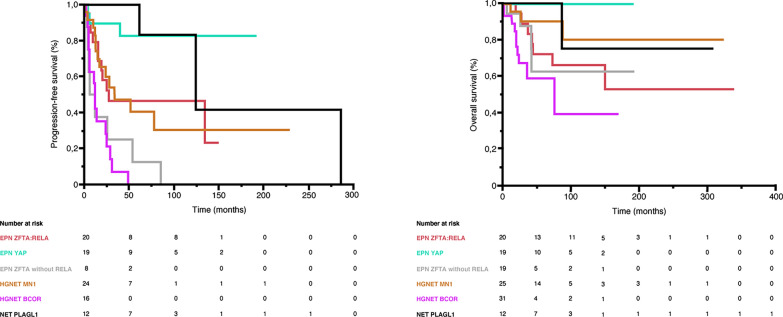
Prognosis for our cases. The mean/median PFS were 70.4/27.6 months for ependymomas, *ZFTA::RELA* fusion-positive, 36.3/not reached months for ependymomas, *YAP1* fusion-positive, 24.4/9.2 months for ependymomas, *ZFTA* non-*RELA* fused, and 43.9/34.0 months for astroblastomas, *MN1*-altered, 16.2/12.0 for CNS tumors with *BCOR* internal tandem duplication and 182.2/277 months for NET PLAGL1 with a significant difference in univariate analysis (p < 0.001). The median OS was not reached for all subgroups except CNS tumors with *BCOR* internal tandem duplication (76.0 months) and the mean OS was not reached for the ependymomas, *YAP1* fusion-positive. The mean OS were 113.5 months for ependymomas, *ZFTA::RELA* fusion-positive, 39.3 months for ependymomas, *ZFTA* non-*RELA* fused, 81.6 months for astroblastomas, *MN1*-altered, 53.2 months for CNS tumors with *BCOR* internal tandem duplication and 111.0 months for NET PLAGL1 with a significant difference in univariate analysis (p = 0.002)

Relevant clinical data are summarized in Supplementary table 1. Median age at diagnosis was 19.0 years (patients’ age ranged from 6 to 40 years). The male/female sex ratio was 0.8 (4 males and 5 females). Tumor location varied; frontal and occipital lobes were the most common locations (6/9 cases, 66%). MRIs were available for 7/9 cases (Fig. 5). The maximal diameter of tumors ranged from 35 to 89 mm. All tumors except one showed a similar imaging pattern: well-demarcated masses, located in the hemispheres with ependymal contact, solid and cystic portions, and variable enhancement after gadolinium-chelate injection. Peritumoral edema was sparse when considering the relatively large tumor size in many cases. The unique exception featured a peripheral cortical location without ependymal involvement, but instead had pachymeningeal contact with scalloping, suggestive of its gradual growth. All patients, except two (cases #1 and #8), underwent gross total resection. None of the patients received adjuvant treatment. Outcome data was available for all patients included in the cohort (Supplementary table 1 for details). We found significant differences in both PFS and OS between the different subgroups in univariate analysis (*p* < 0.001 and p = 0.002, respectively) (Fig. 6).

### Characterization of rare supratentorial subependymomas in young patients

The integrated diagnosis for the six remaining cases of the cohort was supratentorial subependymomas. Indeed, they were less cellular and well-circumscribed from the adjacent brain parenchyma. Tumor cells presented small euchromatic, round to oval nuclei arranged in a fibrillary matrix with microcystic changes and microcalcifications. There were no PAS-positive eosinophilic granular bodies and no hemosiderin deposits. No cases presented necrosis or microvascular proliferation. Mitotic activity and MIB-1 labeling index were low. Using immunohistochemistry, tumor cells diffusely expressed GFAP and showed no or only focal expression of Olig2 and SOX10. A dot-like EMA expression was observed in few tumor cells of one case. No L1CAM immunoreactivity, no nuclear accumulation of NFκB and no expression of neuronal markers were observed. H3K27me3 expression was maintained in all cases. Genetic analyses revealed fusions in three cases: *C1orf194::UQCR10* fusion (cases #13 and #14) and *PDGFB::LRP1* (case #15). DNA-methylation profiling classified two cases (#11 and #12) as supratentorial subependymoma with high calibrated scores (> 0.9). Two other cases (#10 and #14) (with a calibrated score < 0.9) definitively clustered within this MC by t-SNE analysis, despite the inclusion of a case previously identified as spinal ependymoma with the same *C1orf194::UQCR1* fusion (previously reported in [23]). Interestingly, case #13 was identified as enchondromatosis (Ollier-Maffucci syndrome with a germline *IDH2* R172G mutation, also found in the tumor). There was no overexpression/mutation of p53/*TP53* and ATRX expression was maintained.

## Discussion

Ependymomas are currently classified according to their anatomic location (supratentorial, posterior fossa or spinal) and molecular alterations [21]. In the supratentorial area, ependymomas (grade 2 and 3) are subdivided between two genetic subgroups: supratentorial ependymomas, *ZFTA* fusion-positive and *YAP1* fusion-positive, both mainly observed in children [21]. Subependymomas (grade 1) are present in all locations, almost exclusively in adults and without recurrent genetic alterations but have a distinct MC (supratentorial, spinal, and posterior fossa) [21]. Recently, based on DNA-methylation profiling, NET-PLAGL1 were identified as supratentorial pediatric tumors characterized by frequent ependymal or subependymal histological features, and fusions implicating the *PLAGL1* gene [29]. However, because of a wide variety of histopathological findings in this MC, the nosology “NET” was suggested [29]. The current work showed a high proportion (60% of cases) of *PLAGL1* alterations discovered in the selected population of supratentorial ependymomas, non-*ZFTA/*non-*YAP1* fused and supratentorial subependymomas of the young. Histopathological, immunohistochemical (expression of GFAP without Olig2 and SOX10) and ultrastructural findings (particularly, the presence of cilia, junctional apparatuses between the neoplastic glial cells’ intermediate filaments, and microvilli-like structures between tumor cells) [5, 9, 22] were in line with an ependymal differentiation for these tumors. In this series, we identified recurrent histopathological features that can be used as diagnostic determinants of NET-PLAGL1: well-circumscribed tumors with mixed ependymal and subependymoma-like features, calcifications, microcystic changes, siderophages, and coarse eosinophilic granular bodies. Contrary to subependymomas, NET-PLAGL1 seem to be more cellular and may correspond to the tumor type “mixed ependymomas–subependymomas” terminology, found in the current WHO classification [21], which poses a problem for grading and prognosis. Because of limited outcome data, it remains difficult to predict prognosis for patients with NET-PLAGL1 [20, 29, 41, 44]. However, if we compare our data (n = 9) to the literature (n = 3) [20, 41, 44], it seems that NET-PLAGL1 are associated with favorable outcomes in comparison to other supratentorial ependymoma subgroups and other relevant differential diagnoses. Indeed, 11/12 patients were alive at the end of follow-up (median follow-up of 61 months, ranging from 3 to 404 months) [20, 41, 44]. Particularly, five patients were still alive more than 5 years after the initial diagnosis, four of them received no anti-neoplastic treatment other than surgery [20, 41, 44].

Morphologically, the main differential diagnosis is supratentorial ependymoma, *YAP1* fusion-positive [3], however NET-PLAGL1 equally affect males and females, and did not show widespread or strong immunoreactivity for EMA. Because of the inclusion criteria used in the current work, we did not observe a wide variety of histopathological morphologies (except one case with adipocytic metaplasia), but other subtypes of ependymomas may present divergent differentiations [36]. While NET-PLAGL1 was isolated by DNA-methylation profiling, it seems that this MC is not yet stable, as 6/8 tumors with a proven *PLAGL1* alteration were not classified by the v12.5 classifier. When we pooled our cases together with other published cases (n = 52), median age of patients with NET-PLAGL1 was 6.0 (ranging from 0 to 40 years-old, 78% of them were aged less than 18 years-old), with a male:female ratio of 1.3 (29 males and 23 females) [20, 29, 41, 44]. All cases were supratentorial with a predilection for frontal and parietal lobes (44 and 36% of cases) [20, 29, 41, 44]. *PLAGL1* fusion gene partners include: *EWSR1* (56%), *FOXO1* (25%), *EP300* (6%), and for the first time in the current work, *MAML2* (3%) [20, 29, 41, 44]. *MAML2* has previously been reported as a gene partner for fusions in supratentorial ependymomas, *ZFTA* [32, 36] and *YAP1* fusion-positive [35]. In two other NET-PLAGL1, no fusion implicating the *PLAGL1* gene was identified but a chromothripsis of chromosome 6, which contains the *PLAGL1* gene was evidenced (one reported case in [29] and one case in the current work). Finally, genetic analyses failed to reveal any *PLAGL1* or chromosome 6 alterations in the two remaining NET-PLAGL1 (one reported case in [29] and one case in the current work), suggesting that other genes may be implicated or detection limitations in the molecular techniques used. In our study, we evidenced for the first time that FISH analysis to detect a *PLAGL1* rearrangement may constitute a reliable technique for pathology departments not carrying out routine RNA-sequencing or DNA-methylation profiling analyses.

Notably, in the current work, a subset of cases (6/15) was classified as supratentorial subependymomas in young patients, as this diagnosis has been classically observed in patients between 40 and 84 years-old [21]. Genetic features of the reported cases here did not reveal chromosome 19 loss/CNV, or histones’ gene mutations (none of cases from the current series were located on the midline), as previously reported [39, 42]. However, two cases presented a *C1orf194::UQCR10* fusion and one other a *PDGFB::LRP1* fusion. Interestingly, a *C1orf194::UQCR10* fusion was previously reported in one adult case of spinal ependymoma [23]. Using, DNA-methylation and t-SNE analyses, we showed that the three cases with the *C1orf194::UQCR10* fusion did not cluster together, and were classified according to their location (spinal *vs.* supratentorial) and histology (ependymoma *vs.* subependymoma). It has been shown that supratentorial and rare infratentorial forms of ependymomas, *ZFTA* fusion-positive share the same DNA-methylation profiling and are classified within the “ST EPN, ZFTA fusion-positive” MC [15, 33], whereas in ependymal tumors with a *C1orf194::UQCR10* fusion, the epigenetic signature of the tumor location seems predominate in the current DNA-methylation classifier. Further cases with this fusion are needed to better understand if they represent a distinct CNS tumor type or not. One of these cases was described in context of enchondromatosis (with a germline mutation of *IDH2* R172G), which classically predisposes to astrocytomas and oligodendrogliomas [1, 7]. To our knowledge, only one case of ependymoma was previously reported in association with Ollier disease [28]. A *PDGFB::LRP1* has not yet been reported in ependymomas or subependymomas, but only in one low-grade glioma without a definite histological diagnosis [13]. Our case was classified as a subependymoma because it did not express Olig2 and SOX10 markers, which are absent in ependymal tumors [31].

In conclusion, NET-PLAGL1 present histopathological, immunohistochemical and ultrastructural features of an ependymal differentiation, suggestive of a new subclass of supratentorial ependymoma. While they share histological features with other genetic types of supratentorial ependymomas (such as the eosinophilic granular bodies found in *YAP1-*fused cases, and a subset has been found to have the clear cell component seen in *ZFTA-*fused cases), NET-PLAGL1 seem to be an enriched form of the ancient nosology known as “mixed ependymomas–subependymomas”. The tumor’s morphology may incite neuropathologists to suggest this diagnosis and search for *PLAGL1* alteration by RNAseq and /or FISH analysis.

### Supplementary Information

**Additional file 1.**
